# Experiential Avoidance and Bordering Psychological Constructs as Predictors of the Onset, Relapse and Maintenance of Anxiety Disorders: One or Many?

**DOI:** 10.1007/s10608-017-9856-7

**Published:** 2017-05-10

**Authors:** Philip Spinhoven, Albert M. van Hemert, Brenda W. J. H. Penninx

**Affiliations:** 1 Institute of Psychology, Leiden University, Wassenaarseweg 52, 2333 AK Leiden, The Netherlands; 2 Department of Psychiatry, Leiden University Medical Center, Albinusdreef, 2333 ZA Leiden, The Netherlands; 3 Department of Psychiatry/ EMGO Institute for Health and Care Research, VU University Medical Center, AJ Ernststraat 1187, 1081 HL Amsterdam, The Netherlands

**Keywords:** Experiential avoidance, Neuroticism, Rumination, Worry, Anxiety sensitivity, Anxiety, Vulnerability

## Abstract

To investigate (a) the incremental predictive validity of experiential avoidance over and above bordering psychological constructs (i.e., rumination, worry, neuroticism and anxiety sensitivity) in predicting onset, relapse and maintenance of anxiety *disorders*; and (b) whether these related constructs can be represented by a single, higher-order latent factor with similar predictive power as the separate psychological constructs while offering a more parsimonious predictive model. Longitudinal cohort study with repeated assessments after 4 years in a sample of 2157 adults aged 18–65, consisting of 1614 persons with past or current anxiety disorder (Panic Disorder with or without Agoraphobia, Social Anxiety Disorder, Generalized Anxiety Disorder, Agoraphobia without panic) according to the Composite Interview Diagnostic Instrument (CIDI) and 543 controls. Experiential avoidance (Acceptance and Action Questionnaire-I) manifested substantial overlap with bordering cognitive constructs. Experiential avoidance and anxiety sensitivity both uniquely predicted maintenance of anxiety disorders and neuroticism uniquely predicted relapse of anxiety disorders, over and above the effect of the other cognitive constructs. Moreover, a latent factor of psychological vulnerability loaded strongly on each of these psychological constructs. This latent factor predicted onset, maintenance and relapse of anxiety disorders. The tendency to frequently experience strong negative emotions, to evaluate these experiences as aversive and to engage in avoidant coping strategies may constitute a transdiagnostic factor predictive of anxiety disorders. Further developing and testing of interventions targeting transdiagnostic construct underlying anxiety and mood disorders seem warranted.

Experiential avoidance is described as consisting of two related parts: (a) the unwillingness to remain in contact with aversive private experience (including bodily sensations, emotions, thoughts, memories, and behavioral predispositions), and (b) action taken to alter the aversive experiences or the events that elicit them (Hayes et al. 1996). Experiential avoidance as a transdiagnostic construct (Baer 2007; Barlow et al. 2004; Harvey et al. 2004) has been hypothesized to play an important role in the etiology, maintenance and modification of various forms of psychopathology (Hayes 2004), anxiety and depression in particular (for reviews, see Chawla and Ostafin 2007; Hayes et al. 1996).

Recently, Barlow and Kennedy (2016) argued that transdiagnostic concepts proposed for anxiety and mood disorders have in common that they refer to a propensity to find emotional experiences aversive. In particular, individuals with higher levels of neuroticism have a tendency to frequently experience strong negative emotions and to evaluate these experiences as aversive. Such individuals may be more likely to engage in avoidant coping strategies (such as rumination, worry, emotion suppression, experiential avoidance, anxiety sensitivity) to manage their emotions, which paradoxically may increase the frequency/intensity of these negative emotions (cp. Barlow et al. 2014). In a previous study based on data derived from the Netherlands Study of Depression and Anxiety (NESDA), we showed that experiential avoidance predicted onset, relapse as well as maintenance of depressive disorders during a 4-year follow-up period. However, after controlling for rumination, worry and neuroticism, experiential avoidance no longer significantly predicted onset, relapse or maintenance of depressive disorders (Spinhoven et al. 2016). These results suggested that it may be fruitful to study the high interrelatedness of psychological vulnerabilities and their common core rather than psychological vulnerabilities such as experiential avoidance in isolation (Bird et al. 2012; Hong and Cheung 2015).

As experiential avoidance has also been implicated in the onset and development of anxiety, the present study aims to examine the incremental predictive value of experiential avoidance for anxiety disorders, as well as the interrelatedness of experiential avoidance with bordering psychological constructs (i.e., worry, rumination, neuroticism, and anxiety sensitivity). Based on Borkovec’s (1994) seminal theory ascribing an avoidant function to worry, experiential avoidance has been hypothesized to be related to both worry and GAD. In line, cross-sectional studies in non-clinical samples have found a significant and positive relationship of experiential avoidance with pathological worry (Roemer et al. 2005; Ruiz 2014a, b; Santanello and Gardner 2007). Moreover, elevated levels of experiential avoidance in GAD patients compared to non-clinical controls have been observed (Lee et al. 2010; Roemer et al. 2005). In addition, Santanello and Gardner (2007) showed that experiential avoidance mediated the relationship between maladaptive perfectionism and worry. In addition, experiential avoidance was found to mediate the effect of general self-efficacy and anxiety sensitivity on pathological worry (Ruiz 2014a) and the effects of mindfulness skills on pathological worry (Ruiz 2014b).

People with social anxiety disorder may engage in experiential avoidance as well, when avoidance of anxiety provoking social situations is impossible. In agreement with this hypothesis, cross-sectional studies in non-clinical samples indicate that experiential avoidance covaries with symptoms of social anxiety (Glick and Orsillo 2011; Mahaffey et al. 2013). Moreover, experiential avoidance was significantly and positively associated with dysfunctional cognitions related to social comparison and social ineptness, although experiential avoidance did not account for significant additional variance in social anxiety symptoms above and beyond that explained by dysfunctional cognitions (Mahaffey et al. 2013). Experiential avoidance was also significantly and positively associated with self-focused attention and partly mediated the relationship of self-focused attention with severity of social anxiety (Glick and Orsillo 2011). Moreover, in a cross-sectional study of a large sample of help-seeking SAD patients, experiential avoidance predicted impairments in daily life, free time, and social contacts above and beyond dysfunctional attitudes and neuroticism (Gloster et al. 2011). Finally, it has been found that the effect of anxiety sensitivity and behavioral inhibition on concurrent social anxiety disorder was not moderated by level of experiential avoidance (Panayiotou et al. 2014).

For panic disorder, empirical evidence for a relation with experiential avoidance has been obtained in biological challenge experiments (e.g., breathing carbon dioxide-enriched air inducing panic symptoms). Among healthy participants, those with higher levels of experiential avoidance reported more panic symptoms and greater distress following the challenge procedure (Feldner et al. 2003; Karekla et al. 2004; Spira et al. 2004). Moreover, participants with panic disorder or a history of panic attacks manifested higher levels of experiential avoidance than controls, also after controlling for level of depressive symptoms (Baker et al. 2004; Tull and Roemer 2007). Anxiety sensitivity has been studied as a cognitive construct that could explain the relation of experiential avoidance with panic disorder. Both constructs relate to a person’s reactions to symptoms, but anxiety sensitivity refers exclusively to anxiety, while experiential avoidance refers to a person’s unwillingness to experience negatively evaluated thoughts and emotions more generally. Experiential avoidance and anxiety sensitivity have been found to be positively associated in non-clinical samples (Zvolensky and Forsyth 2002), but the physical concerns dimension of anxiety sensitivity predicted concurrent anxiety symptoms over and above experiential avoidance both in a student and a patient sample (Berman et al. 2010; Wheaton et al. 2010). In patients with panic disorder with agoraphobia, experiential avoidance and anxiety sensitivity were overlapping yet distinct constructs with experiential avoidance explaining additional variance in scores for anticipatory anxiety and panic-related disability but not in panic attacks, agoraphobic avoidance and health worries (Kämpfe et al. 2012). Study results on experiential avoidance as a mediating or moderating variable in the relation of anxiety sensitivity with anxiety are inconclusive (Bardeen et al. 2013, 2014; Kashdan et al. 2006, 2008).

Of note is that most of the studies of experiential avoidance and bordering psychological constructs in anxiety are cross-sectional using non-clinical samples. A fundamental limitation of cross-sectional studies is that such a design precludes concluding whether cognitive constructs are risk factors (i.e., a correlate shown to precede outcome) or can better be seen as correlates, signs and symptoms, concomitants or even consequences. Moreover, none of the available longitudinal studies of experiential avoidance examined presence of anxiety disorder instead of symptom severity as outcome variable. An exception is a previous longitudinal study (Spinhoven et al. 2014) showing that experiential avoidance may be conceptualized as a transdiagnostic factor affecting the course and development of comorbidity of anxiety and depressive disorders. However, this study did not examine the specific relationship of experiential avoidance with onset, relapse and maintenance of anxiety disorders and also did not consider the predictive role of experiential avoidance in relationship to bordering psychological constructs.

The primary aim of the present *longitudinal* study is to assess the incremental predictive validity of experiential avoidance over and above bordering psychological constructs (i.e., rumination, worry, neuroticism and anxiety sensitivity) in predicting onset, relapse and maintenance of anxiety *disorders*. Moreover, given their relatively high intercorrelations it will be investigated whether these constructs can be represented by a single, higher-order latent factor (Hong and Cheung 2015) with similar predictive power as the separate psychological constructs while offering a more parsimonious predictive model for the onset, relapse and maintenance of anxiety disorders.

## Methods

### Sample

The Netherlands Study of Depression and Anxiety (NESDA) is an ongoing cohort study designed to investigate determinants, course and consequences of depressive and anxiety disorders. The NESDA sample of 2981 adults (18–65 years) includes participants with a lifetime and/or current anxiety and/or depressive disorder (n = 2329; 78%) and controls (persons without depressive or anxiety disorders; n = 652; 22%). To include various developmental stages of disorders and different levels of severity, participants were recruited from general practices (n = 1610; 54%), mental health organizations (n = 807; 27%), and the general population (n = 564; 19%). General exclusion criteria were a primary diagnosis of severe psychiatric disorders such as psychotic, obsessive compulsive, bipolar or severe addiction disorder, and not being fluent in Dutch. A detailed description of the NESDA design and sampling procedures has been given elsewhere (Penninx et al. 2008). The research protocol was approved by the Ethical Committees of the participating universities and all respondents provided written informed consent.

The baseline assessment included demographic and personal characteristics, a standardized diagnostic psychiatric interview, an extensive set of psychological measures and a medical assessment including blood sampling. After 2 (T2), 4 (T4), and 6 years (T6) a face-to-face follow-up assessment was conducted with a response of 87.1% (n = 2596) at T2, of 80.6% (n = 2402) at T4 and of 75.7% (n = 2256) at T6. Experiential avoidance was measured at T2 for the first time and consequently the T2 measurement constituted the baseline measurement in the present study.

For the purpose of the present study we selected the following three subgroups: (a) persons with a 6-month recency anxiety disorder at T2, that could persist during the follow-up period from T2 to T6 (n = 711; anxious group); (b) persons with a history of previous anxiety disorders but no 6-month recency anxiety disorder at T2, who could have a relapse during the follow-up (n = 903; previously anxious group); and (c) persons with no history of previous anxiety or depressive disorders and no 6-month recency anxiety or depressive disorder at T2, who could develop an anxiety disorder during the follow-up (n = 543; non-anxious group).

### Measures

#### Psychiatric Diagnosis

Past and present DSM-IV (APA 1994) depressive [Major Depressive Disorder (MDD), Dysthymia (DYS)] and anxiety [Panic Disorder with or without Agoraphobia (PD), Social Anxiety Disorder (SAD), Generalized Anxiety Disorder (GAD), Agoraphobia without panic (AGO)] disorders at T2, T4 and T6 were assessed using the Composite Interview Diagnostic Instrument (CIDI, version 2.1). The CIDI is used worldwide and WHO field research has found high interrater reliability (Wittchen et al. 1991), high test–retest reliability, (Wacker et al. 2006) and high validity for depressive and anxiety disorders (Wittchen et al. 1989; Wittchen 1994). The CIDI was administered by more than 40 research assistants that have been trained, including psychologists, nurses or residents in psychiatry. Research assistants received 1 week of training by the fieldwork coordinator, and were certified to conduct assessments following approval of audiotapes of at least two complete interviews. Question wording and probing behavior of interviewers was constantly monitored by checking a random selection of about 10% of all taped interviews. In addition, a continuous monitoring system of interviewer variances and interviewer specific item-non response was maintained through computer analyses in SPSS software.

Maintenance was defined as any anxiety disorder (PD, SAD, GAD or AGO) between T2 and T6 in persons with a 6-month recency anxiety disorder at T2. Relapse was defined as any anxiety disorder between T2 and T6 in persons with no 6-month recency anxiety disorder at T2, but with a history of previous anxiety disorders. Onset was defined as any anxiety disorder between T2 and T6 in persons with no 6-month recency depressive or anxiety disorder at T2 and in addition no lifetime history of previous depressive or anxiety disorders.

#### Anxiety

Severity of anxiety and behavioral avoidance at T2 was measured with the Beck Anxiety Inventory (BAI; Beck et al. 1988; Dutch version; Beck and Steer 2015), respectively the Fear Questionnaire (FQ; Marks and Mathews 1979). The BAI is a 21-item self-report questionnaire to measure severity of generalized anxiety and panic symptoms in particular. This scale has shown sound psychometric properties such as factorial validity, internal consistency, and test–retest stability, as well as adequate convergent and divergent validity (Osman et al. 2002). The FQ is a 15-item self-report scale to measure external avoidance behavior (for example, ‘travelling alone or by bus’ or ‘speaking or acting to an audience’) and has been proven valid and reliable in a Dutch population (Van Zuuren 1988). Internal consistency of the BAI in the present study was 0.92 and of the FQ 0.89.

#### Experiential Avoidance

Experiential avoidance at T2 was assessed using the Dutch version of the 9-item Acceptance and Action Questionnaire-I (AAQ-I; Hayes et al. 2004). A previous study of the Dutch AAQ-I showed that a one-factor model, with AAQ items constituting a single dimension of experiential avoidance, fitted the data well. Also the internal consistency (0.74) and temporal stability of the AAQ (0.82) were satisfactory (Boelen and Reijntjes 2008). Internal consistency of the AAQ-I in the present study was 0.69.

#### Worry

Worry at T2 was measured with the Dutch version of the Penn State Worry Questionnaire (PSWQ; Meyer et al. 1990; Van Rijsoort et al. 1999). The PSWQ consists of two subscales: a ‘General worry’ subscale (11 items) and a ‘Not-worry’ subscale (5 items) (Van Rijsoort et al. 1999). The ‘General worry’ subscale accounts for most of the variance in PSWQ scores (Meyer et al. 1990; Van Rijsoort et al. 1999), and only this subscale was administered in the NESDA study. The PSWQ has been proven to be a valid measure of trait worrying unaffected by the content of the worry (Davey 1993) with high internal consistency, good test–retest reliability and unaffected by social desirability (Meyer et al. 1990). Internal consistency of the General worry scale in the present study was 0.96.

#### Rumination

Rumination at T2 was assessed using the 6-item subscale Rumination on Sadness (RUM) of the revised version of the Leiden Index of Depression Sensitivity (LEIDS-R; Van der Does 2002; Williams et al. 2008). Participants are asked to indicate whether and how their thinking patterns change when they experience mild dysphoria. LEIDS-RUM scores are significantly associated with scores for rumination on the Ruminative Response Scale (RRS) after controlling for current depressive symptoms, showing that the observed relationship is independent of current mood state (Moulds et al. 2008). In the present sample the internal consistency of the RUM-scale was 0.84.

#### Neuroticism

Neuroticism at T2 was measured with the subscale for neuroticism of the Dutch version of the 60-item NEO Five-Factor Inventory (NEO-FFI; Costa and McCrae 1995, 1992; Dutch version; Hoekstra et al. 1996). The NEO-FFI questionnaire measures the following five personality domains: Neuroticism, Extraversion, Agreeableness, Conscientiousness and Openness to Experience. Internal consistency values range from 0.74 to 0.89. Cronbach’s alpha’s of the neuroticism subscale in the present study was 0.81.

#### Anxiety Sensitivity

Anxiety Sensitivity at T2 was measured using the Anxiety Sensitivity Index −16 items (Peterson and Reiss 1992; Reiss et al. 1986). This self-report questionnaire indicates the degree to which respondents are concerned about possible negative consequences of anxiety-related sensations. Items are scored on a 5-point Likert scale ranging from ‘0 = very little’ to ‘4 = very much’. Total scores range from 0 to 64. The ASI has high levels of internal consistency, good test–retest reliability, and good validity (Peterson and Plehn 1999; Reiss et al. 1986). The internal consistency of the total scale in this study was good: α = 0.89.

### Statistical Analyses

As we pursued to assess the predictive value of psychological constructs for onset, relapse and maintenance of anxiety disorders, all statistical analyses described below were conducted separately in the non-anxious, previously anxious and anxious group.

First, univariable logistic regression analyses were conducted to assess the predictive value of T2 sociodemographic (i.e., age, gender, and education), clinical (i.e., severity of anxiety and avoidance behavior and co-morbid depressive disorder (MDD and/or DYS)), and psychological variables (i.e., experiential avoidance, rumination, worry, anxiety sensitivity and neuroticism) for presence of anxiety disorders during the follow-up period between T2 and T6. Next, two multivariable models were constructed, one model including all sociodemographic and clinical variables (Model 1A) and one model including all psychological variables (Model 1B). Finally, in order to determine which psychological variables were independent risk factors for presence of anxiety disorders during the follow-up period over and above sociodemographic and clinical variables both models were combined (Model 2A). By definition, presence of comorbid depressive disorders was not included as a covariate in the non-anxious group.

Secondly, we investigated the fit of a single higher order latent factor of psychological vulnerability with T2 experiential avoidance, worry, rumination, anxiety sensitivity and neuroticism scores as indicators using Confirmatory Factor Analysis (CFA). Next, we examined the predictive value of this latent factor of psychological vulnerability for onset, relapse and maintenance of anxiety disorders (Model 1C) also after controlling for sociodemographic and clinical variables (Model 2B).

CFA and logistic regression using latent factor scores were performed using MPlus v.7.1 (Muthén and Muthén 1998–2012). All other statistical analyses were run using SPSS version 21 (IBM Corp. 2012). A significance level of p < .05 was used for all analyses.

## Results

### Sample Characteristics

Of the 2157 participants selected at T2, 1793 completed the T6 measurements. Study dropouts (n = 364; 16.9%) between T2 and T6 did not differ from study completers (n = 1429; 83.1%) regarding age, gender and level of rumination. However, in comparison to completers dropouts were significantly less educated and showed higher levels of anxiety symptoms, behavioral avoidance, experiential avoidance, worry and neuroticism as well as a higher proportion of persons with multiple anxiety disorders or comorbid depressive disorders. However, the effect sizes for the differences between both groups were negligible (Cohens’d <0.2 or Cramer’s phi <0.10).

Table 1 shows sociodemographic, clinical and psychological characteristics of our three subgroups at T2: (a) persons with a 6-month recency anxiety disorder at T2 (n = 711; anxious group); (b) persons with no 6-month recency anxiety disorder at T2, but with a history of previous anxiety disorders (n = 903; previously anxious group); and (c) persons with no 6-month recency anxiety or depressive disorder at T2 and also no history of previous anxiety or depressive disorders (n = 543; non-anxious group). As can be derived from this table, level of anxiety and behavioral avoidance, experiential avoidance, rumination, worry, anxiety sensitivity and neuroticism significantly differed between groups with anxious participants scoring higher than previously anxious participants, who scored higher than non-anxious participants on each of these variables. The prevalence of 6-month recency anxiety disorders in the disordered group at T2 was as follows: SAD: n = 359 (50.5%); PD: n = 290 (40.8%); GAD: n = 194 (27.3%); and AGO: n = 147 (20.7%). Of the disordered participants at T2, 474 (66.7%) had a single anxiety disorder, 195 (27.4%) had two anxiety disorders, and 42 (5.9%) had three anxiety disorders. Table 2 shows the correlations among the psychological constructs and severity of anxiety and behavioral avoidance at T2. As can be derived from this table, all psychological constructs are significantly intercorrelated with effect sizes ranging from 0.46 (anxiety sensitivity with rumination) to 0.76 (worry with neuroticism).

**Table 1:** Overview of sociodemographic, clinical and psychological characteristics of the three subgroups

	1. Anxious group (n = 711)		2. Previously anxious group (n = 903)		3. Non-anxious group (n = 543)		Overall statistics Χ^2^ (df)/F (df) p value	Contrasts
	M	SD	M	SD	M	SD		
Age	41.8	12.4	42.3	12.7	41.3	14.6	1.00 (2) ns	1 = 2 = 3
Female gender (n/%)	487	68.5	621	68.8	329	60.6	11.88 (2)*	1 = 2 > 3
Years of education	11.7	3.3	12.2	3.2	12.9	3.2	21.05 (2)*	1 < 2 < 3
BAI	15.7	9.9	8.0	7.1	3.0	3.8	439.57 (2)*	1 > 2 > 3
FQ	33.9	20.0	17.9	14.7	9.1	9.0	401.94 (2)*	1 > 2 > 3
MDD/DYS	366	51.5	169	18.7	0	0	629.75 (1)*	1 > 2
AAQ-I	38.2	6.7	33.0	6.5	26.6	6.0	439.80 (2)*	1 > 2 > 3
LEIDS: RUM	11.1	5.0	8.3	4.7	4.0	3.6	347.38 (2)*	1 > 2 > 3
PSWQ	36.7	10.2	28.7	10.6	18.2	7.4	497.68 (2)*	1 > 2 > 3
NEO-FFI: N	40.6	7.2	33.8	7.6	25.5	6.6	646.29 (2)*	1 > 2 > 3
ASI	32.8	9.4	27.1	7.9	22.3	4.6	258.93 (2)*	1 > 2 > 3

*BAI* Beck Anxiety Inventory, *FQ* Fear Questionnaire, *MDD* Major Depressive Disorder, *DYS* Dysthymia, *AAQ-I* Acceptance and Action Questionnaire-I, *LEIDS:RUM* Leiden Index of Depression Sensitivity-Revised: Rumination on Sadness subscale, *PSWQ* Penn State Worry Questionnaire; *NEO-FFI:N* NEO Five-Factor Inventory: Neuroticism subscale, *ASI* Anxiety Sensitivity Index

*p < .001

**Table 2:** Correlations of psychological constructs and symptom severity

Variables	1	2	3	4	5	6	7
1. AAQ-I	(−)						
2. LEIDS:RUM	0.58	(−)					
3. PSWQ	0.65	0.65	(−)				
4. NEO-FFI: N	0.69	0.63	0.76	(−)			
5. BAI	0.53	0.50	0.58	0.64	(−)		
6. FQ	0.52	0.46	0.53	0.61	0.60	(−)	
7. ASI	0.54	0.47	0.55	0.50	0.56	0.50	(−)

All p < .001; *AAQ-I* Acceptance and Action Questionnaire-I, *LEIDS:RUM* Leiden Index of Depression Sensitivity-Revised: Rumination on Sadness subscale, *PSWQ* Penn State Worry Questionnaire, *NEO-FFI:N* NEO Five-Factor Inventory: Neuroticism subscale, *BAI* Beck Anxiety Inventory, *FQ* Fear Questionnaire, *ASI* Anxiety Sensitivity Index

### Common Core Psychological Vulnerabilities

As all psychological constructs showed relatively high intercorrelations, using MPlus we performed an Exploratory Factor Analysis (EFA) with the oblique rotation of GEOMIN and maximum likelihood as estimation method. A one-factor model representing experiential avoidance, worry, rumination, neuroticism, and anxiety sensitivity as separate but correlated indicators showed a good fit to the data [CFI = 0.98; TLI = 0.96; RMSEA = 0.08 (90% CI 0.07–0.10)]. Each of the psychological constructs had a strong and significant relationship with the latent variable: experiential avoidance = 0.77, worry = 0.87, rumination = .72, neuroticism = 0.86, and anxiety sensitivity = 0.62. EFA with two factors resulted in a Heywood case due to negative residual variance of the ASI, probably as the result of overfactoring. In the non-fitting two-factor solution without fit parameters and standard errors, only the ASI had a high loading on a second factor. As we were unable to directly compare differences in fit between a one and two-factor model using MPlus, we decided to additionally conduct a Principal Component Analysis (PCA) using SPSS. Results showed that there was only one component with an Eigenvalue >1 (i.e., 3.44) explaining 68.80% of the variance in cognitive constructs. Loadings on the single factor ranged from 0.64 to 0.87 (worry = 0.87; neuroticism = 0.85; experiential avoidance = 0.79; rumination = .74; anxiety sensitivity = 0.64). The pattern matrix of a two-factor solution also showed that only the ASI loaded on a separate factor. On the basis of these converging results of the EFA and PCA analyses, we concluded that a one-factor solution is appropriate and decided to use latent factor scores of psychological vulnerability with experiential avoidance, worry, rumination, neuroticism, and anxiety sensitivity as indicators in our further MPlus analyses.

### Prediction of Anxiety Disorders During T2-T6 by Psychological Constructs at T2

In the non-anxious group (n = 484; attrition = 11.9%) the incidence of anxiety was 4.4%, in the previously anxious group (n = 742; attrition = 17.8%) the relapse rate was 34.6% and in the anxious group (n = 603; attrition = 15.2%) 72.5% of the persons had persisting anxiety disorder during the follow-up period. Univariable logistic regression analyses showed that each of the psychological constructs predicted onset of anxiety disorders during T2-T6 (see Univariable Model in Table 3), as well as relapse of anxiety disorders during T2-T6 (see Univariable Model in Table 4), and maintenance of anxiety disorders during T2-T6 (see Univariable Model in Table 5). After controlling for other psychological constructs, none of the individual psychological constructs remained predictive of onset of anxiety disorders (see Model 1B in Table 3), only neuroticism remained predictive of relapse of anxiety disorders (OR = 1.77; 95% CI 1.33–2.37; see multivariable model 1B in Table 4), and both experiential avoidance (OR = 1.44; 95% CI 1.04–1.98) and anxiety sensitivity (OR = 1.45; 95% CI 1.16–1.83) remained predictive of maintenance of anxiety disorders (see multivariable model 1B in Table 5). After additionally controlling for sociodemographic and clinical characteristics, only relapse of anxiety disorders was predicted by experiential avoidance (OR = 1.38; 95% CI 1.05–1.82) (see multivariable model 2A in Table 4), while the OR of rumination became <1, most likely because of multicollinearity among predictor variables.

**Table 3:** Sociodemographic, clinical and psychological predictors of onset of anxiety disorders in the non-anxious group with follow-up data (n = 776)

	Univariable Model 1^a^		Multivariable Model 1^b^		Multivariable Model 2^c^			
	OR	95% CI	Model 1A		Model 2A		Model 2B	
			OR	95% CI	OR	95% CI	OR	95% CI
Sociodemographic characteristics								
Age	0.98	0.95–1.01	0.98	0.95–1.01	0.98	0.95–1.02	0.99	0.96–1.02
Gender	2.86	0.95–8.63	2.14	0.68–6.68	2.09	0.65–6.75	1.97	0.61–6.31
Education	0.87	0.76–1.01	0.90	0.77–1.04	**0.82**	**0.68–0.98**	0.86	0.73–1.00
Clinical characteristics								
BAI (per SD increase)	**3.53**	**1.76–7.10**	**2.35**	**1.01–5.54**	1.24	0.44–3.46	1.28	0.48–3.44
FQ (per SD increase)	**2.85**	**1.48–5.48**	1.82	0.83–3.97	1.01	0.40–2.55	1.07	0.43–2.67
			Model 1B					
Psychological constructs								
AAQ-I (per SD increase)	**2.40**	**1.34–4.30**	1.24	0.60–2.56	1.06	0.50–2.27		
LEIDS:RUM (per SD increase)	**2.48**	**1.43–4.13**	1.43	0.72–2.85	1.75	0.83–3.69		
PSWQ (per SD increase)	**3.08**	**1.85–5.10**	1.55	0.70–3.46	1.46	0.64–3.36		
NEO-FFI:N (per SD increase)	**3.07**	**1.73–5.42**	1.55	0.68–3.51	1.18	0.49–2.82		
ASI (per SD increase)	**2.85**	**1.58–5.13**	1.57	0.74–3.35	2.08	0.90–4.79		
			Model 1C					
Latent psychological factor			**15.40**	**4.34–54.67**			**10.53**	**2.10–52.89**

Significant results are depicted in bold

^a^Based on univariable logistic regression

^b^Based on multivariable logistic regression with all sociodemographic and clinical variables (Model 1A) or all psychological variables (Model 1B) in the model

^c^Based on multivariable logistic regression with all sociodemographic, clinical and psychological variables in the model

*BAI* Beck Anxiety Inventory, *FQ* Fear Questionnaire, *AAQ-I* Acceptance and Action Questionnaire-I, *LEIDS:RUM* Leiden Index of Depression Sensitivity-Revised: Rumination on Sadness subscale, *PSWQ* Penn State Worry Questionnaire, *NEO-FFI:N* NEO Five-Factor Inventory: Neuroticism subscale, *ASI* Anxiety Sensitivity Inventory

**Table 4:** Sociodemographic, clinical and psychological predictors of relapse of anxiety disorders in the previously anxious group with follow-up data (n = 742)

	Univariable Model 1^a^		Multivariable Model 1^b^		Multivariable Model 2^c^			
			Model 1A		Model 2A		Model 2B	
	OR	95% CI	OR	95% CI	OR	95% CI	OR	95% CI
Sociodemographic characteristics								
Age	**0.99**	**0.98–0.99**	**0.98**	**0.97–0.99**	**0.98**	**0.97–0.99**	**0.99**	**0.97–0.99**
Gender	1.22	0.87–1.70	1.16	0.81–1.66	1.27	0.86–1.89	1.25	0.85–1.84
Education	0.99	0.94–1.03	1.01	0.96–1.06	1.05	0.99–1.11	1.02	0.97–1.08
Clinical characteristics								
BAI (per SD increase)	**1.77**	**1.45–2.15**	**1.42**	**1.14–1.78**	1.25	0.98–1.61	**1.30**	**1.02–1.65**
FQ (per SD increase)	**2.13**	**1.75–2.60**	**1.83**	**1.48–2.28**	**1.72**	**1.34–2.20**	**1.72**	**1.35–2.18**
MDD/DYS	**1.73**	**1.19–2.51**	1.21	0.80–1.84	0.94	0.59–1.50	0.92	0.58–1.46
		Model 1B						
Psychological constructs								
AAQ-I (per SD increase)	**1.73**	**1.42–2.11**	1.28	0.99–1.66	**1.38**	**1.05–1.82**		
LEIDS:RUM (per SD increase)	**1.23**	**1.04–1.46**	**0.78**	**0.62–0.98**	**0.71**	**0.56–0.91**		
PSWQ (per SD increase)	**1.60**	**1.33–1.92**	1.12	0.86–1.45	1.09	0.83–1.44		
NEO-FFI:N (per SD increase)	**2.04**	**1.66–2.50**	**1.77**	**1.33–2.37**	1.35	0.98–1.86		
ASI (per SD increase)	**1.42**	**1.20–1.68**	1.15	0.94–1.41	1.06	0.85–1.32		
			Model 1C					
Latent psychological factor			**2.99**	**2.09–4.29**			**1.72**	**1.12–2.63**

Significant results are depicted in bold

^a^Based on univariable logistic regression

^b^Based on multivariable logistic regression with all sociodemographic and clinical variables (model 1A) or all psychological variables (model 1B) in the model

^c^Based on multivariable logistic regression with all sociodemographic, clinical and psychological variables in the model

*BAI* Beck Anxiety Inventory, *FQ* Fear Questionnaire, *MDD* Major Depressive Disorder, *DYS* Dysthymia, *AAQ-I* Acceptance and Action Questionnaire-I, *LEIDS:RUM* Leiden Index of Depression Sensitivity-Revised: Rumination on Sadness subscale, *PSWQ* Penn State Worry Questionnaire, *NEO-FFI:N* NEO Five-Factor Inventory: Neuroticism subscale, *ASI* Anxiety Sensitivity Inventory

**Table 5:** Sociodemographic, clinical and psychological predictors of maintenance of anxiety disorders in the anxious group with follow-up data (n = 603)

	Univariable Model 1^a^		Multivariable Model 1^b^		Multivariable Model 2^c^			
			Model 1A		Model 2A		Model 2B	
	OR	95% CI	OR	95% CI	OR	95% CI	OR	95% CI
Sociodemographic characteristics								
Age	1.00	0.99–1.02	0.99	0.98–1.01	1.00	0.98–1.02	1.00	0.98–1.02
Gender	0.87	0.59–1.30	0.85	0.55–1.30	0.99	0.62–1.59	0.88	0.55–1.38
Education	**0.93**	**0.88–0.98**	0.95	0.89–1.01	**0.93**	**0.88–0.99**	**0.94**	**0.88–0.99**
Clinical characteristics								
BAI (per SD increase)	**1.85**	**1.51–2.27**	**1.59**	**1.26–2.01**	**1.54**	**1.17–2.02**	**1.50**	**1.16–1.94**
FQ (per SD increase)	**1.91**	**1.56–2.33**	**1.59**	**1.28–1.99**	**1.63**	**1.27–2.10**	**1.59**	**1.25–2.02**
MDD/DYS	1.21	0.89–1.73	0.78	0.52–1.17	0.75	0.47–1.18	0.69	0.44–1.07
		Model 1B						
Psychological constructs								
AAQ-I (per SD increase)	**1.76**	**1.40–2.22**	**1.44**	**1.04–1.98**	1.37	0.98–1.92		
LEIDS:RUM (per SD increase)	**1.32**	**1.07–1.62**	0.99	0.78–1.27	1.03	0.80–1.34		
PSWQ (per SD increase)	**1.56**	**1.25–1.95**	1.08	0.79–1.47	1.09	0.79–1.50		
NEO-FFI:N (per SD increase)	**1.49**	**1.18–1.87**	1.07	0.75–1.51	0.75	0.50–1.11		
ASI (per SD increase)	**1.69**	**1.37–2.08**	**1.45**	**1.16–1.83**	1.17	0.91–1.50		
			Model 1C					
Latent psychological factor			**2.33**	**1.65–3.29**			1.39	0.91–2.12

Significant results are depicted in bold

^a^Based on univariable logistic regression

^b^Based on multivariable logistic regression with all sociodemographic and clinical variables (model 1A) or all psychological variables (model 1B) in the model

^c^Based on multivariable logistic regression with all sociodemographic, clinical and psychological variables in the model

*BAI* Beck Anxiety Inventory, *FQ* Fear Questionnaire, *MDD* Major Depressive Disorder, *DYS* Dysthymia, *AAQ-I* Acceptance and Action Questionnaire-I, *LEIDS:RUM* Leiden Index of Depression Sensitivity-Revised:Rumination on Sadness subscale, *PSWQ* Penn State Worry Questionnaire, *NEO-FFI:N* NEO Five-Factor Inventory:Neuroticism subscale, *ASI* Anxiety Sensitivity Inventory

Using logistic regression in Mplus, anxiety disorder during the follow-up period was regressed on latent factor scores of psychological vulnerability with experiential avoidance, worry, rumination, neuroticism, and anxiety sensitivity as indicators. Psychological vulnerability significantly predicted onset of anxiety disorders during T2–T6 (OR = 15.40; 95% CI 4.34–54.67; see Model 1C in Table 3), relapse of anxiety disorders during T2–T6 (OR = 2.99; 95% CI 2.09–4.29; see Model 1C in Table 4), and maintenance of anxiety disorders during T2–T6 (OR = 2.33; 95% CI 1.65–3.29; see Model 1C in Table 5). After additionally controlling for sociodemographic and clinical characteristics, latent factor scores remained predictive of onset of anxiety disorders (OR = 10.53; 95% CI 2.10–52.89; see multivariable model 2B in Table 4), and relapse of anxiety disorders (OR = 1.72; 95% CI 1.12–2.63; see multivariable model 2B in Table 4). In predicting onset of anxiety disorders, the latent factor of psychological vulnerability was the only significant predictor, while relapse of anxiety disorders was also significantly predicted by younger age and higher levels of anxiety and behavioral avoidance. Lower education and higher levels of anxiety and behavioral avoidance also significantly predicted maintenance of anxiety disorders and the latent factor of psychological vulnerability did not significantly improve upon this prediction.

## Discussion

Our first research question was whether experiential avoidance is an independent, overlapping or proxy risk factor of onset, relapse, or maintenance of anxiety disorders. In accordance with extant literature and a previous NESDA study (Spinhoven et al. 2016), experiential avoidance was significantly associated with rumination (Cribb et al. 2006; Giorgio et al. 2010; Morina 2011), worry (Roemer et al. 2005; Ruiz 2014a, b; Santanello and Gardner 2007), anxiety sensitivity (e.g., Berman et al. 2010; Kämpfe et al. 2012; Wheaton et al. 2010; Zvolensky and Forsyth 2002) and neuroticism (Boelen and Reijntjes 2008; Bond and Bunce 2003; Hayes et al. 2004; Kashdan et al. 2006). The moderately strong interrelationships among psychological constructs indicated that experiential avoidance cannot be considered to constitute an independent risk factor and that the predictive value of experiential avoidance for anxiety disorders has to be considered together with associated psychological constructs.

Consequently, we examined how experiential avoidance worked together with bordering psychological constructs (i.e. rumination, worry, anxiety sensitivity and neuroticism) as an overlapping or proxy risk factor of anxiety disorders. Assessed separately experiential avoidance at baseline predicted onset, relapse and maintenance of anxiety disorders during the 4-year follow-up period. After controlling for the other psychological constructs at baseline, experiential avoidance and anxiety sensitivity remained predictive of maintenance of anxiety disorders, while only neuroticism predicted relapse of anxiety disorders and none of the psychological constructs independently predicted onset of anxiety disorders. After additionally controlling for sociodemographic and clinical risk factors, experiential avoidance significantly predicted relapse of anxiety disorders. These results diverge from a previous NESDA study (Spinhoven et al. 2016) which showed that experiential avoidance could best be conceptualized as a proxy risk factor for depressive disorders as repetitive negative thinking in the form of rumination and worry constituted more dominant risk factors (Olatunji et al. 2013), while in the present study repetitive negative thinking in the form of rumination and worry was no independent predictor neither for onset, nor relapse or maintenance of anxiety disorders. The present study suggests that in the context of anxiety disorders however experiential avoidance as an overlapping risk factor uniquely predicts relapse and maintenance of anxiety disorders, although its predictive value for maintenance was no longer significant after additionally controlling for severity of anxiety and behavioral avoidance. However, these conclusions have to be interpreted with caution as conclusions about what processes are better predictors are based on small differences between interrelated psychological processes all associated with onset, relapse and maintenance of anxiety disorders in univariable analyses.

Our second research question was whether the psychological constructs investigated may be largely redundant with one another and could be represented by a single, higher-order latent factor with similar predictive power as the separate psychological constructs while offering a more parsimonious predictive model for the onset, relapse and maintenance of anxiety disorders. In most studies, a single psychological construct has been studied in relation to a specific disorder making it difficult to assess whether these putative individuating and unique constructs provide incremental information across psychological constructs and disorders. Notwithstanding the theoretical importance of such an approach, factor analyses in the present study showed that a single latent factor for psychological vulnerability loaded strongly onto all five measured cognitive processes with a good fit to the data. Moreover, this factor of psychological vulnerability showed a comparable or stronger relationship with onset, relapse and maintenance of anxiety disorders than the five psychological processes individually. These results concur with those of a recent meta-analysis of six cognitive constructs (i.e., pessimistic inferential style, dysfunctional attitudes, ruminative style, anxiety sensitivity, intolerance of uncertainty, and fear of negative evaluation) relevant for depression and anxiety reporting that all loaded on a single underlying factor (Hong and Cheung 2015).

This finding of a single latent factor underlying all cognitive constructs suggests that they share a common core and is consistent with the notion of a transdiagnostic etiologic process that underlies anxiety and depressive disorder. Barlow and Kennedy (2016) recently argued that the core psychopathological mechanism or functional relationship in anxiety and mood disorders consists of intense negative emotional reactions as manifested by individuals with higher levels of neuroticism and subsequent efforts to down-regulate these aversive negative emotional experiences. Persons with anxiety and depressive disorders respond with greater distress to their own emotional experience (e.g., Brown and Barlow 2009), have more difficulties in accepting their emotions (e.g., Tull and Roemer 2007) and are less tolerant of their negative emotions (e.g., Roemer et al. 2005). As a result, they are inclined to down-regulate these negative emotional experiences (e.g., Aldao et al. 2010). As experiential avoidance, rumination, worry, and anxiety sensitivity can be conceptualized as transdiagnostic concepts referring to this propensity to find emotional experiences aversive, the latent factor loadings on these constructs and neuroticism may represent a crucial higher-order latent factor of psychological vulnerability for anxiety and mood disorders.

An alternative interpretation of a latent psychological vulnerability factor is provided by dispositional-trait theories of psychopathology (e.g., Clark et al. 1994) stating that genetically based dispositions like neuroticism constitute a broad and undifferentiated vulnerability to the development of anxiety and mood disorders with cognitive constructs such as anxiety sensitivity as mediating variables between these broad-distal dispositions and specific-proximal forms of psychopathology (e.g., Hong and Paunonen 2011). In this view the common core would primarily reflect neuroticism as associated with the other cognitive constructs.

The notion of a transdiagnostic etiologic process underlying anxiety and depressive disorder also bears clinical relevance as it suggests that treatment protocols that target putative transdiagnostic etiologic processes could be clinically useful. Results of a recent meta-analysis of psychological transdiagnostic treatments for anxiety and mood disorders in adults yield preliminary evidence that transdiagnostic interventions in comparisons with disorder-specific treatments are as effective for reducing anxiety, and may be superior for reducing depression (Newby et al. 2015). These promising findings could potentially curtail the proliferation of treatment manuals for different disorders (Craske 2012) and help treating persons with comorbid disorders in a more unified way.

Interestingly, the size of the predictive value of the latent factor of psychological vulnerability was the largest for onset of anxiety disorder (OR = 4.41) (while symptom level of anxiety and behavioral avoidance were no significant predictors yet) and the smallest for maintenance of anxiety disorder (OR = 1.76) (while after correcting for much higher symptom levels of anxiety and behavioral avoidance the latent factor was no longer a significant predictor). These results suggest that psychological vulnerability better signals future incidence of anxiety disorders in the absence of symptoms of anxiety and avoidance. Alternatively, indices of psychological vulnerability such as worry or fear of anxiety symptoms may be non-specific prodromal symptoms occurring before more specific anxiety symptoms become prominent.

The ‘shrinking’ predictive value of psychological vulnerability in the prediction of relapse and maintenance of anxiety disorders may be due to various reasons. A simple statistical reason could be that these characteristics are associated with severity of anxiety and avoidance and that by statistically controlling for these variables, which almost define the presence of anxiety disorder, the statistical power of psychological variables to uniquely predict anxiety disorder is reduced. Another possibility is that although these psychological characteristics contribute to a person “crossing the line” from feeling anxious into an anxiety disorder, once an individual has acquired an anxiety disorders other self-perpetuating processes emerge that determine chronicity. Behavioral factors such as avoidance may constitute important maintaining processes (Hendriks et al. 2013). Excessive use of behavioral or psychological avoidance behavior is considered as a maladaptive coping style that may impede control or habituation of anxiety and in this way contribute to chronicity (Barlow 2002).

As argued previously (Spinhoven et al. 2016) we think that alternative moderation or mediation models to explain how experiential avoidance and other individual psychological risk factors work together in predicting anxiety and mood disorders might be less appropriate. The MacArthur approach (Kraemer et al. 2008, 2001) defines strict eligibility criteria to identify whether a variable is a candidate for consideration as a potential moderator (or mediator) based on association and temporal precedence. More specifically this approach stipulates (a) that a moderator must precede another psychological construct as focal predictor and that moderator and predictor are independent (moderation) and (b) that another psychological construct as a predictor must precede experiential avoidance as a mediator and that predictor and mediator are associated (mediation).

Given the on average high intercorrelations between psychological constructs, moderation models may not be warranted. Also in the present study experiential avoidance and related psychological constructs proved to be substantially interrelated precluding further moderation analyses. Not meeting the criteria of independence and temporal precedence for moderation analysis may help to explain the inconsistent results of previous cross-sectional (Bardeen et al. 2013; Kashdan et al. 2008; Panayiotou et al. 2014) and prospective studies of experiential avoidance as a moderating variable in anxiety (Bardeen et al. 2014).

According to the MacArthur approach, the high intercorrelations between psychological constructs, however, makes the applicability of mediation models more likely. However, regarding psychological constructs such as anxiety sensitivity, rumination, worry, neuroticism and experiential avoidance it is impossible to state that some of these constructs constitute more “fundamental” characteristics appearing earlier in development preceding a putative mediating variable (Kraemer et al. 2001). Which of the constructs is measured first, which is entered into a regression analysis first, or which has greater predictive value does not establish temporal precedence. These considerations seriously question the conclusions on experiential avoidance as a mediating variable in previous cross-sectional studies in anxiety (e.g., Glick and Orsillo 2011; Kashdan et al. 2006; Ruiz 2014a; Santanello and Gardner 2007) (as in cross-sectional studies temporal precedence of the predictor is absent by definition).

The present study has some notable strengths: first, a longitudinal cohort study in a large representative sample of participants with various anxiety disorders from different recruitment settings; second, use of a structured diagnostic interview to assess presence of anxiety disorders; third, analyzing experiential avoidance as an independent, overlapping, or proxy risk factor for onset, relapse and maintenance of anxiety disorders; and fourth analyzing experiential avoidance as an indicator of psychological vulnerability together with bordering psychological constructs.

The results of this study need to be considered in light of several limitations. First, the AAQ-I as used in the present study has been criticized for having overly complex items or items showing overlap with other concepts (Chawla and Ostafin 2007). A new 7-item AAQ-II has been developed to assess the same construct as the AAQ-I in order to improve its psychometric consistency (Bond et al. 2011). However, the correlation of the AAQ-I with the AAQ-II is very high (r = .97) (Bond et al. 2011), suggesting that both versions measure comparable constructs although the psychometric consistency of the AAQ-II is somewhat better. Second, as the NESDA study was not specifically designed to answer the present research questions, some relevant measures of transdiagnostic risk factors for anxiety and mood disorders (such as deficits in mindfulness, and negative appraisals and attributions reflecting the neurotic sense of uncontrollability) were not available. Third, given the relatively high comorbidity among depressive and anxiety disorders, the differential effects of experiential avoidance and related psychological constructs on anxiety disorders may have been biased by comorbid depressive disorders, although we statistically controlled for the presence of comorbid depressive disorders in our analyses. Fourth, we had to compile participants with different anxiety disorders into one group, because there were not enough patients per single anxiety disorder diagnosis (e.g. pure GAD) to allow for separate analyses. Fifth, although attrition was relatively low and there were only negligible differences in demographic, clinical and psychological variables between study drop-outs and study completers, attrition may restrict generalizability of our findings and may have resulted in biased estimates of associations among study variables. Sixth, many NESDA participants recruited in primary and specialized care received medication (39.1%), formal psychoeducation or -therapy, counseling or skills training (44.0%) or both (21.4%) before or during the study period. However, treatment was not a significant determinant of the 2-year course of anxiety and depression in multivariate analyses also containing clinical information on e.g. severity and duration of symptoms (Penninx et al. 2011). Treatment may well influence the course of the disorders, but since clinical indicators also determine receipt of treatment, an observational study may end up finding no association. It is therefore unlikely that treatment received was an effect modifier of the relation of psychological predictors with course of anxiety.

## Conclusions

Experiential avoidance, although predictive of onset, relapse and maintenance of anxiety disorders in univariable analyses, only proved to be an independent risk factor for maintenance of anxiety disorders after controlling for bordering psychological constructs. Neuroticism, experiential avoidance, rumination, worry, and anxiety sensitivity proved to be highly interrelated and can be seen as indicators of a latent factor representing a tendency to frequently experience strong negative emotions, to evaluate these experiences as aversive and to engage in avoidant coping strategies. As this latent factor predicted onset and relapse of anxiety disorders even after controlling for sociodemographic and clinical severity variables, further developing and testing of interventions targeting transdiagnostic constructs underlying anxiety and mood disorders seem warranted (Hong and Cheung 2015).
